# Multidisciplinary perinatal management of paroxysmal kinesigenic dyskinesia in pregnancy: a case report and a proposed structured pathway

**DOI:** 10.3389/fmed.2026.1795479

**Published:** 2026-04-20

**Authors:** Weimin Ding, Junqin Mao, Minling Chen, Zhimin Sheng

**Affiliations:** 1 Department of Obstetrics and Gynecology, Wenling Maternal and Child Health Care Hospital, Taizhou, China; 2 Department of Anesthesiology, Wenling Maternal and Child Health Care Hospital, Taizhou, China

**Keywords:** cesarean delivery, multidisciplinary care, paroxysmal kinesigenic dyskinesia, perinatal management, pregnancy

## Abstract

**Background:**

Paroxysmal kinesigenic dyskinesia (PKD) is a rare movement disorder characterized by brief dystonic or dyskinetic attacks precipitated by sudden voluntary movement or arousal. Pregnancy complicated by PKD is extremely rare, and evidence guiding perinatal decision-making, especially regarding delivery mode and anesthetic management remains scarce.

**Case presentation:**

We report a term pregnancy in a woman with long-standing PKD managed through a multidisciplinary team involving neurology, obstetrics, anesthesiology, and neonatology. Following individualized optimization of antiseizure medications (ASMs) and intensive antenatal surveillance, the patient showed moderate symptomatic improvement during pregnancy. Given the high risk of pain, stress, or movement-triggered dystonic attacks with transient loss of voluntary motor control during labor, elective cesarean delivery was planned. Combined spinal–epidural anesthesia (CSEA) was performed to provide effective analgesia while minimizing arousal and sensory triggers. The procedure was uneventful, with no perioperative dystonic episodes. A healthy neonate was delivered with no congenital anomalies.

**Conclusion:**

This case demonstrates that favorable maternal and neonatal outcomes can be achieved in pregnancies complicated by PKD through individualized antiseizure medication management, multidisciplinary care, and trigger-oriented perinatal planning. The structured clinical decision-making pathway proposed in this study may provide a practical framework for managing this rare and challenging condition.

## Introduction

Paroxysmal kinesigenic dyskinesia (PKD) is a rare movement disorder characterized by recurrent, brief episodes of involuntary hyperkinetic movements, most commonly choreoathetosis or dystonia, typically triggered by sudden voluntary movement or arousal. The global prevalence is estimated to be 1 in 150,000 individuals, with a male predominance reported in sporadic cases (1, 2). Although the underlying pathophysiology remains incompletely understood, pathogenic variants in *PRRT2* have been identified as the most common genetic cause, with *TMEM151A* recently recognized as an additional disease-associated gene, highlighting the genetic heterogeneity of PKD (3–8).

PKD usually manifests during childhood or adolescence and may persist into adulthood. While the disorder is generally considered benign, frequent attacks can significantly impair quality of life and functional independence (9, 10). Most published studies have focused on adolescent or non-pregnant adult populations; however, clinical evidence regarding pregnancy-associated PKD is extremely limited. The interaction between pregnancy and PKD is complex, as hormonal fluctuations, altered neuronal excitability, and fetal safety concerns related to antiseizure medications (ASMs) may modulate disease activity and shape perinatal decision-making. In particular, labor-associated factors such as pain, emotional stress, and abrupt positional changes may act as potent triggers for dystonic episodes, posing unique challenges for obstetric care.

An extensive literature search identified only five publications describing a total of 19 pregnancy cases with PKD or related paroxysmal dyskinesias (Table 1). PKD was the predominant subtype (18/19), whereas paroxysmal non-kinesigenic dyskinesia (PNKD) was reported in only one case. The *PRRT2 c.649dupC* variant was the most frequently identified pathogenic mutation, while genetic data were unavailable in most patients. During pregnancy, symptoms improved or remitted in the majority of cases, with exacerbation rarely reported. ASMs were used selectively based on individualized risk–benefit considerations, and obstetric outcomes were generally favorable, with only one reported cesarean delivery and no major maternal or fetal complications (1, 11–14). However, detailed multidisciplinary perinatal management strategies, including anesthetic planning, trigger-oriented delivery decision-making, and structured perinatal workflows remain scarce, and no standardized guidance currently exists.

**Table 1:** Main characteristics of pregnant patients with PKD and related paroxysmal dyskinesias identified in literature review.

Authors	Patients reported	Age at onset	Trigger	Attack phenomenology	Gene	Medication	Symptoms during pregnancy	Pregnancy outcome
Madeja et al. (1999) (12)	1	11	Sudden change of body posture	PKD, choreoathetoid attacks	Not reported	Phenytoin	Remission of attacks	Not reported
Fabbri et al. (2013) (11)	1	9	Sudden voluntary movements	PKD, choreoathetoid-dystonic attacks	PRRT2 c.649dupC (p.Arg217Profs*8)	Carbamazepine (200 mg/die)	Amelioration of symptoms	Not reported
Fabbri et al. (2013) (11)	1	10	Sudden voluntary movements	PKD, choreoathetoid attacks	PRRT2 c.649dupC (p.Arg217Profs*8)	Triptans for concurrent migraine	Amelioration of symptoms	Not reported
Fabbri et al. (2013) (11)	1	12	Sudden voluntary movements	PKD, choreoathetoid-dystonic attacks	PRRT2 c.649dupC (p.Arg217Profs*8)	Not reported	Remission of attacks	Not reported
Bovenzi et al. (2020) (14)	1	15	Sudden voluntary movements	PKD, dystonia (right-side limbs, head rotation, facial dyskinesia)	PRRT2 c.649dupC (p.Arg217Profs*8)	Lamotrigine (100–120 mg/day)	Amelioration of symptoms	Cesarean delivery at 39 + 2 weeks
Bruno et al. (2004) (1)	7	Childhood–adolescence	Sudden voluntary movements	PKD, involuntary movements	Not reported	Not reported	Amelioration of symptoms	Not reported
Bruno et al. (2004) (1)	1	Childhood–adolescence	Sudden voluntary movements	PKD, involuntary movements	Not reported	Not reported	Worsening of symptoms	Not reported
Bruno et al. (2004) (1)	5	Childhood–adolescence	Sudden voluntary movements	PKD, involuntary movements	Not reported	Not reported	Not reported	Not reported
Friedman et al. (2009) (13)	1	Childhood	Anxiety, coffee/alcohol intake	PNKD, dystonia (painful spasms)	MR-1 variant (not available)	Diazepam, clonazepam	Completely remission	Not reported

PKD, paroxysmal kinesigenic dyskinesia; PNKD, paroxysmal non-kinesigenic dyskinesia.

Given the rarity of PKD in pregnancy and the potential intrapartum risks posed by movement, pain, and stress-induced dystonic attacks, individualized and coordinated management is essential. Here, we report a case of term pregnancy complicated by PKD that was successfully managed via a coordinated multidisciplinary team (MDT) approach involving neurology, obstetrics, anesthesiology, and neonatology. Based on this experience and a literature review of existing literature, we propose a structured perinatal management pathway to guide clinical decision-making and address the current gap in the literature.

## Case presentation

**Figure 1:**
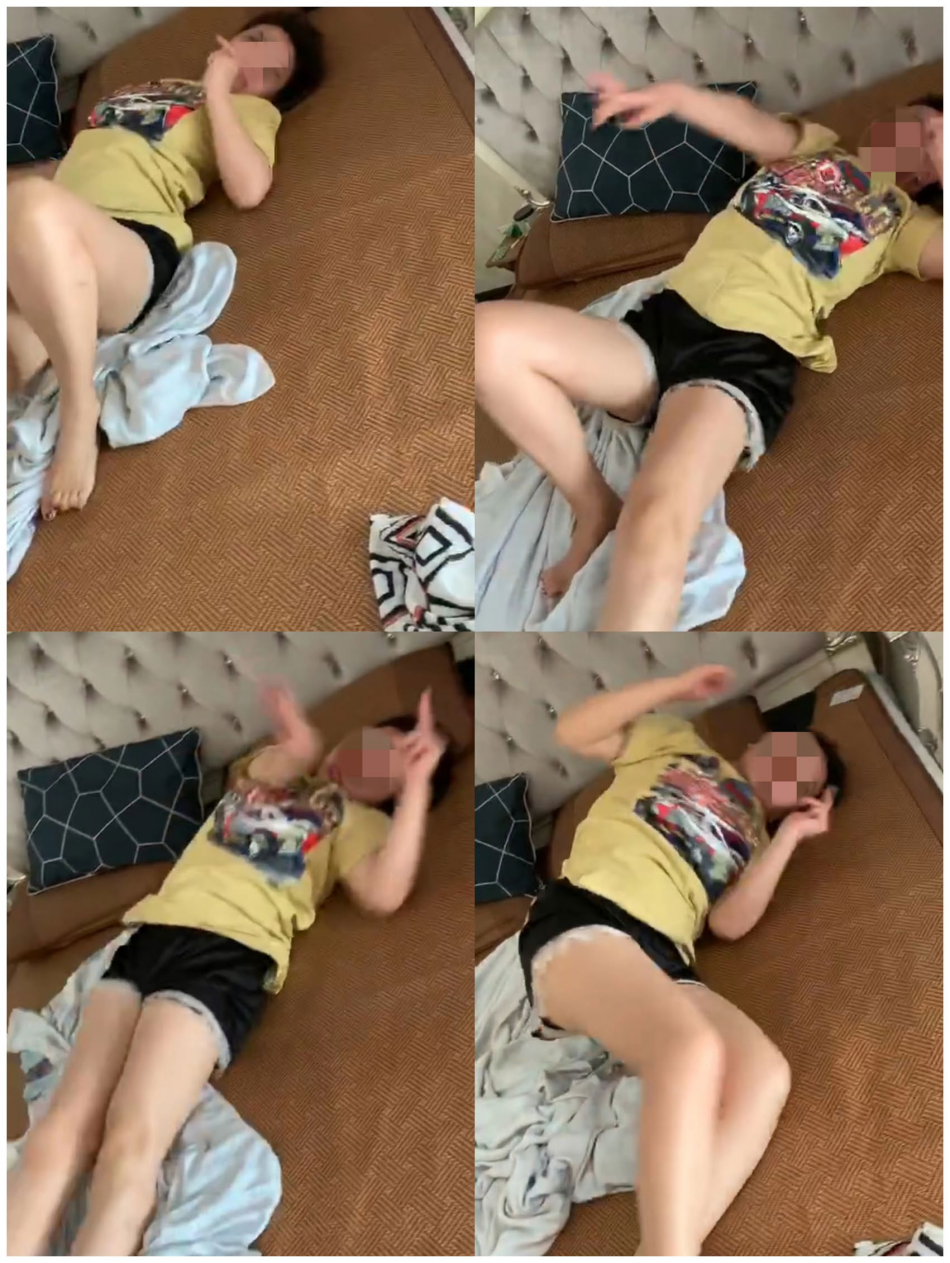
This figure shows an attack that occurred after the patient woke up at approximately 20 weeks of pregnancy. The clinical features included sudden arousal-induced dance-like movements of the torso and limbs, dystonia, and other dyskinetic manifestations, with no loss of consciousness. The duration was approximately 1 min.

A woman in her early 30s (gravida 4, para 0) was admitted at 37 + 1 weeks of gestation for perinatal management of PKD. Her symptoms began at approximately 1 year of age and evolved into recurrent, brief episodes of involuntary choreiform and dystonic movements involving the limbs and trunk, occasionally accompanied by facial dyskinesia, without impairment of consciousness. Attacks were typically triggered by sudden awakening or voluntary movement and resolved spontaneously within 1–2 min (Figure 1).

Extensive neurological evaluations, including electroencephalography, brain magnetic resonance imaging, sleep studies, and genetic testing revealed no structural or epileptiform abnormalities. Primary (idiopathic) PKD was diagnosed based on typical clinical features and exclusion of major secondary causes. Long-term treatment consisted of carbamazepine (200 mg once daily [QD] orally), haloperidol (6 mg QD orally), and trihexyphenidyl (6 mg QD orally). Perampanel (2 mg once daily) was added 2 years prior to conception. Before pregnancy, attack frequency was approximately 10–20 episodes per day.

Upon confirmation of pregnancy, perampanel was discontinued due to limited safety data during pregnancy. Carbamazepine was replaced with oxcarbazepine (300 mg QD orally), and the doses of haloperidol and trihexyphenidyl were reduced by 50% under neurological supervision. During pregnancy, the patient reported a moderate reduction in attack frequency compared with the pre-pregnancy period, with approximately 5–10 episodes per day, each lasting less than one minute.

Serial antenatal evaluations, including nuchal translucency screening, oral glucose tolerance testing, detailed fetal ultrasonography, and fetal echocardiography at 24 weeks of gestation, were unremarkable. Fetal echocardiography confirmed a normal four-chamber view, normal great vessel outflow tracts, and no structural heart defects. Maternal blood pressure, glucose levels, and fetal growth were all within normal ranges. Thyroid function tests (total triiodothyronine [T3], thyroxine [T4], free triiodothyronine [FT3], free thyroxine [FT4], thyroid-stimulating hormone [TSH]), serum electrolytes, and thyroid ultrasound were all normal.

Given the rarity and complexity of PKD in pregnancy, multidisciplinary care involving obstetrics, neurology, anesthesiology, neonatology, and genetic counseling was implemented. Genetic counseling was provided by a clinical geneticist. Although PKD is often inherited in an autosomal dominant manner with incomplete penetrance, targeted genetic testing for PRRT2 and TMEM151A was negative, leaving the causative variant in this family unidentified. Therefore, targeted prenatal genetic testing was not feasible, and non-invasive prenatal testing for PKD-specific mutations was not pursued. Invasive prenatal diagnostic procedures were discussed; however, considering the absence of a confirmed pathogenic variant and the associated risk of procedure-related miscarriage, the patient and her family declined such testing, and no invasive prenatal procedures were performed.

**Figure 2:**
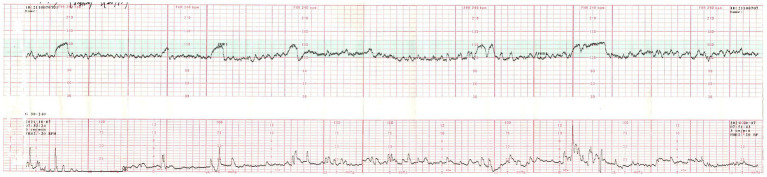
A 20-min FHR monitoring during the morning awakening attack showed a reactive NST, with a baseline FHR of 120 beats per minute (bpm). No fetal distress was detected, and no uterine contractions were induced.

On admission, maternal vital signs were stable. Neurological examination revealed bilaterally absent lower limb tendon reflexes, normal muscle strength (Grade V) in the left lower limb, increased tone in the bilateral ankle dorsiflexors, unstable and inaccurate truncal rotation to the left, and a positive Romberg sign. Obstetric examination confirmed a singleton fetus in left occipitoanterior position with a baseline fetal heart rate (FHR) of 130 beats/min. Continuous FHR monitoring during a typical morning attack demonstrated a reactive non-stress test (NST), with no uterine contractions or signs of fetal compromise (Figure 2).

**Figure 3:**
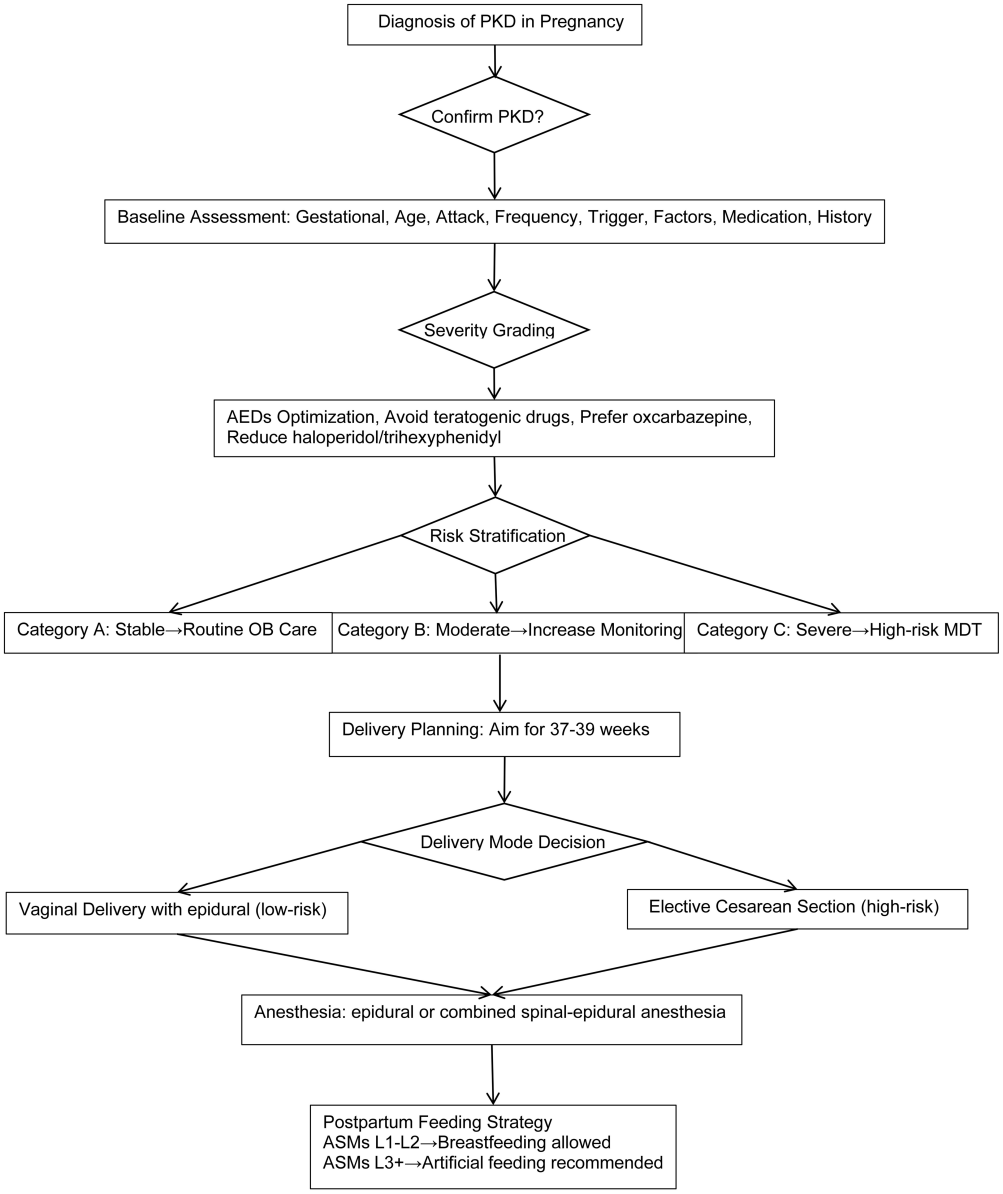
Proposed clinical management flowchart for pregnancy complicated by PKD. [Diagnosis → Severity grading (based on attack frequency and functional impact) → ASM optimization (balancing maternal control and fetal safety) → Risk stratification (trigger sensitivity and disease stability) → Delivery mode selection (integration of neurological and obstetric risks) → Anesthesia plan (minimization of trigger exposure) → Postpartum monitoring (monitor symptoms and adjust lactation medications safely)]. This is a proposed evidence-informed pathway for clinical reference, not a formal guideline.

Given that sudden movement, pain, and emotional stress were consistent triggers for dystonic attacks in this patient, and that episodes were associated with transient loss of voluntary motor control, the MDT determined that a trial of labor posed significant intrapartum risks, including maternal injury, hyperventilation, and interrupted fetal monitoring. In accordance with the structured perinatal management pathway proposed in this study (Figure 3), elective lower-segment transverse cesarean delivery under combined spinal–epidural anesthesia (CSEA) was scheduled.

The patient remained fully awake throughout the procedure to avoid arousal-related triggers, and adequate analgesia was achieved without excessive sensory stimulation. The surgery was uneventful, with an estimated blood loss of 300 mL. No intraoperative dystonic episodes occurred. A healthy male neonate weighing 3,175 g was delivered, with an Apgar score of 10 at 1 and 5 min. No congenital anomalies were detected.

On postoperative day 2, the patient experienced recurrence of dystonic episodes at a frequency and severity similar to her preoperative baseline. Postpartum follow-up confirmed a return to her prepregnancy symptom pattern. Formula feeding was recommended because haloperidol and trihexyphenidyl are classified as L3 drugs during lactation.

This case report was approved by the institutional ethics committee, and written informed consent was obtained from the patient. The report was prepared in accordance with the CARE guidelines (15).

## Discussion

This case provides a comprehensive illustration of the complex interaction between PKD, pregnancy-related neurophysiological changes, and perinatal clinical decision-making. Beyond reporting a favorable maternal and neonatal outcome, this study offers mechanistic insights and a structured multidisciplinary management framework for a condition that remains poorly characterized in obstetric practice.

Although PKD is most frequently associated with pathogenic variants in *PRRT2* and, more recently, *TMEM151A*, approximately 60–70% of clinically diagnosed patients lack identifiable genetic mutations, including the present case (6, 8, 16, 17). This highlights that PKD remains primarily a clinical diagnosis grounded in characteristic clinical features and trigger patterns rather than molecular confirmation alone. The absence of a genetic marker does not imply a benign phenotype but rather reflects the current limits of molecular characterization in paroxysmal movement disorders.

Available evidence suggests that PKD symptoms tend to ameliorate during pregnancy (11, 13, 14). Consistently, the patient in the present case also exhibited a moderate reduction in attack frequency during pregnancy. This phenomenon may be attributed to elevated levels of pregnancy-associated neuroactive steroids, such as progesterone and allopregnanolone, which can enhance neuronal network stability by potentiating GABAergic inhibition or regulating sodium channel function (18, 19). However, the potential relationship between PKD and hormonal status remains insufficiently understood. Importantly, symptom improvement during pregnancy does not necessarily indicate a reduced intrapartum risk. Labor involves intense nociceptive stimulation, emotional stress, autonomic activation, and abrupt positional changes, all of which may serve as potent triggers for dystonic attacks in patients with PKD. Therefore, pregnancy should be viewed as a dynamic state in which baseline neuronal excitability may decrease, while situational trigger susceptibility during labor may paradoxically increase.

Similar to epilepsy and other paroxysmal movement disorders, PKD involves neuronal hyperexcitability and shares overlapping management principles. ASMs may raise fetal risks depending on drug type, dosage, polytherapy, and gestational timing. Therefore, ASMs should be individualized during pregnancy to balance teratogenic risk and maternal symptom control (20). Furthermore, relevant triggers should be minimized during labor, and an MDT approach should be adopted throughout the perinatal period. Although existing epilepsy and movement disorder guidelines cannot be fully extrapolated to PKD in pregnancy, they provide a valuable theoretical framework for clinical decision-making.

The optimal delivery mode for women with PKD remains controversial due to the absence of formal PKD-specific obstetric guideline. For patients with frequent, unpredictable dystonic attacks and impaired voluntary motor control, vaginal delivery carries substantial risks, whereas elective cesarean delivery enables predictable scheduling and tailored anesthetic management. We therefore propose that PKD represents a trigger-sensitive neurological condition, and delivery mode should be determined by combined obstetric and neurological risk stratification rather than conventional obstetric indications alone. Notably, only 1 of 19 previously reported PKD pregnancy cases described cesarean delivery under epidural anesthesia, with detailed anesthetic strategies largely missing in the literature. Our case provides one of the few comprehensive accounts of structured intrapartum care supported by trigger-oriented risk assessment, as well as detailed insights into medication adjustment, delivery mode selection, and anesthetic planning, which have been rarely addressed in prior studies (1, 11–14). The multidisciplinary team fully evaluated the feasibility of vaginal delivery with epidural analgesia, but ultimately opted for elective cesarean delivery for three key reasons: first, pain, stress, and abrupt positional changes during labor increase acute dystonic attack risk, potentially causing loss of motor control, maternal injury, hyperventilation, and unreliable fetal monitoring; second, epidural analgesia cannot fully eliminate stress and arousal triggers; third, the patient’s frequent attacks (5–10 episodes per day) would severely interfere with labor monitoring and fetal safety assessment. We suggest that vaginal delivery with epidural analgesia and close monitoring may be attempted in PKD patients with low attack frequency, intact motor function, and mild trigger sensitivity, whereas elective cesarean delivery is a safer alternative for high-risk patients with frequent attacks, impaired coordination, and prominent pain or stress-related triggers.

From an anesthetic perspective, neuraxial anesthesia was pathophysiologically justified in this case. Unlike epilepsy anesthesia which prioritizes preventing convulsive seizures and cerebral hypoxia, anesthetic management for PKD focuses on minimizing sensory stimulation and abrupt positional changes. CSEA provided rapid, stable analgesia while preserving consciousness and minimizing sensory overload. Adequate analgesia is critical because nociceptive stimuli are well-established triggers for PKD attacks. Avoiding general anesthesia also reduces the risk of abrupt arousal and autonomic instability. No perioperative dystonic episodes occurred under optimized CSEA, supporting the feasibility of this approach.

Unlike prior reports that mainly focused on pregnancy outcomes, this study is, to our knowledge, one of the few to propose a structured multidisciplinary perinatal pathway for PKD in pregnancy, integrating neurology, obstetrics, anesthesiology, and neonatology. This report also includes comprehensive perioperative neurological monitoring, fetal heart rate assessment during PKD attacks, and systematic postpartum follow-up, all of which were insufficiently documented in previous studies.

The clinical decision-making pathway for PKD in pregnancy proposed herein is not based solely on this single case, but constructed from three sources: (1) the experience and consensus of our institutional multidisciplinary team in managing high-risk pregnancies and pregnancies complicated by neurological disorders; (2) a literature review of published PKD pregnancy cases (Table 1); and (3) established principles from the Chinese Expert Consensus on PKD and relevant pregnancy guidelines for other paroxysmal neurological disorders such as epilepsy (20, 21). However, given the current limitations in available evidence, the proposed flowchart (Figure 3) is not intended to serve as a formal guideline but rather as a practical, evidence-informed framework for clinical reference. The key steps are rationally justified as follows: Severity grading was based on attack frequency, motor impairment, and trigger burden, adapted from non-pregnant PKD severity criteria for pregnancy-specific intrapartum risk assessment (1). Medication optimization was guided by epilepsy management principles in pregnancy to balance maternal symptom control and fetal safety (22). Delivery mode selection was based on a two-dimensional assessment of trigger-related risk and motor functional status to appropriately stratify candidates for vaginal delivery or cesarean delivery. Anesthetic management favored neuraxial techniques (epidural or CSEA) for effective analgesia while avoiding loss of consciousness and autonomic instability linked to general anesthesia. Postpartum care focuses on monitoring symptom recurrence and safe medication adjustment compatible with lactation.

Several limitations warrant acknowledgment. First, conclusions are derived from a single case, limiting generalizability despite the rarity of PKD in term pregnancy; future multi-center registries are needed to accumulate larger cohorts. Additionally, not all metabolic and endocrine causes of secondary paroxysmal dyskinesia were fully excluded, as parathyroid hormone (PTH), calcitonin, and ceruloplasmin were not evaluated. Second, the proposed mechanism that neuroactive steroids underlie pregnancy-related symptom improvement remains hypothetical, since serial hormonal and neurophysiological assessments were not conducted to provide direct evidence. Third, the anesthetic strategy was individualized rather than comparatively evaluated. Although CSEA was effective, the lack of a control group precludes conclusions regarding the relative superiority of different anesthetic techniques. Comparative observational studies are needed to inform anesthetic best practices in PKD. Finally, postpartum follow-up was limited to early neurological outcomes. Long-term maternal disease course, breastfeeding-related medication adjustments, and offspring neurodevelopment were not systematically assessed. Longer-term follow-up is needed to evaluate sustained maternal and child outcomes.

## Conclusion

Although PKD complicated by term pregnancy is rare, favorable maternal and neonatal outcomes can be achieved through individualized, trigger-oriented, and multidisciplinary perinatal management. Optimization of antiseizure medications, enhanced antenatal surveillance, and delivery planning tailored to disease-specific triggers are essential components of care. Elective cesarean delivery under neuraxial anesthesia represents a reasonable option for carefully selected patients at high risk of intrapartum dystonia. Importantly, the structured clinical decision-making pathway proposed in this study may provide a practical framework to support clinicians in managing this rare but challenging condition.

## Data Availability

The original contributions presented in the study are included in the article/supplementary material, further inquiries can be directed to the corresponding author.
